# Editorial: Insights in molecular diagnostics and therapeutics: 2021

**DOI:** 10.3389/fmolb.2022.1014416

**Published:** 2022-10-21

**Authors:** Matteo Becatti, William C. Cho

**Affiliations:** ^1^ Department of Experimental and Clinical Biomedical Sciences “Mario Serio”, University of Firenze, Firenze, Italy; ^2^ Department of Clinical Oncology, Queen Elizabeth Hospital, Kowloon, Hong Kong SAR, China

**Keywords:** biomedical research, molecular diagnostic, therapeutics, biomarkers, precision medicine

The most recent advances and discoveries in biomedical research, together with technological progress, have made a paradigm shift from traditional medicine towards more precise and predictable health care, customized for an individual patient at a specific time. In this era of precision medicine, having validated biomarkers to inform clinical decision-making is crucial. Biomarkers have various applications including disease diagnosis and monitoring, prognosis, and prediction of response to treatment. This Research Topic collects some of the latest molecular diagnostic and therapeutic advances and their studies in precision medicine.

The interplay between basic and applied research is essential for the study of the molecular and cellular mechanisms that regulate biological systems. The correlation between the integrated “omics” data, such as the genome, transcriptome, proteome, and other “omics” information, should be considered in a patient-specific, rather than symptom-specific, approach with precision medicine (Abdelhalim et al., 2022). This approach must be adapted to each individual’s unique omics leading to personalized management of diseases. While most existing studies analyze the omics data separately, data integration is crucial on the horizon of precision medicine by using machine learning or artificial intelligence approaches (Hasanzad et al., 2021). However, the use of “omics” technologies generates enormous amounts of data that require complex computational analyses and interpretations. In this Research Topic, five original research articles (Jiang et al.; Cao et al.; Sun et al.; Wang et al.; Yuan et al.) presented the interesting “omics” studies that identify new diagnostic and prognostic biomarkers using bioinformatic tools and public databases in diverse types of cancer. Technologies such as array-based hybridization assays and next-generation sequencing promote the identification of new molecular targets and the development of prognostic and predictive biomarkers for their use in precision medicine (Wang and Zheng, 2021). Sorokin et al. used public databases to compare and validate gene signatures of microsatellite instability status by RNA-Seq as diagnostic biomarkers in solid tumors. Moreover, RNA expression-based signature is used by Gudkov et al. to predict sorafenib response in kidney cancer. The identification of new prognostic and predictive biomarkers is essential for cancer drug development and therapeutic decision-making for individual patients. One of the greatest challenges today is to develop prognostic and predictive biomarkers that translate the genomic information of individual tumors into a personalized therapeutic approach. In this regard, Zou et al. investigated the role of Toll-like receptor (TLR) subtypes expression in kidney renal clear cell carcinoma (KIRC). Bioinformatics analysis and experimental data demonstrate that the occurrence and development of KIRC are closely related to TLRs and TLRs can be early diagnostic and prognostic biomarkers of KIRC. In another way, Wang et al. explored the diagnostic and prognostic values of long non-coding RNA AP000695.2 in lung adenocarcinoma. Using a data mining approach in The Cancer Genome Atlas and the Gene Expression Omnibus databases, they demonstrated that a higher expression of AP000695.2 correlated with aggressive clinicopathological characteristics and AP000695.2 was an independent prognostic indicator for the overall survival, disease-free survival, and progression-free survival in patients with lung adenocarcinoma. Bioinformatics methods are also used by Zhang et al. to study the impacts of N6-methyladenosine (m^6^A) methylated mRNAs on epigenetic changes after myocardial infarction. In this Research Topic, another interesting retrospective study analyzes new potential biomarkers of idiopathic pulmonary fibrosis (IPF) by mRNA-Seq analysis of IPF lung tissue obtained from surgical lung biopsy and lung transplantation (Qian et al.). Through bioinformatics analyses using Gene Ontology and Kyoto Encyclopedia of Genes and Genomes public databases, the authors identified differentially expressed transcripts, suggesting the synergic role of extracellular matrix remodeling, lipid metabolism, and immune effects in the early development of IPF.

Indeed, bioinformatic tools are essential for data management in modern biology, life sciences, and medicine. In an interesting work, Zhang et al. through metagenomic next-generation sequencing and bioinformatics analysis, explored the changes in the lung microbiome before and after treatment in acute respiratory distress syndrome (ARDS) patients demonstrating the key role of respiratory tract microbiome in the pathogenesis and development of ARDS.

Last but not the least, Mannucci et al. reviewed the recent advances in the role of oxidative stress in the pathogenesis of male infertility, underlining the clinical use of redox biomarkers and the new treatment of oxidative-stress-mediated male infertility.

Together, the articles comprising this Research Topic shed some light on the current status of molecular diagnostic and therapeutic advances. Each report raises questions and indicates aspects that require further attention and scientific inquiry. The progress of molecular diagnostics will continue to grow in the race to enhance care for individuals using genomic and metagenomic information followed by artificial intelligence data evaluation and machine learning algorithms. This approach should combine traditional clinical data with patients’ biological profiles including various omics-based datasets to create a new and exciting path of personalized medicine.
